# Modeling of amorphous SiC_x_O_6/5_ by classical molecular dynamics and first principles calculations

**DOI:** 10.1038/srep42705

**Published:** 2017-02-14

**Authors:** Ningbo Liao, Miao Zhang, Hongming Zhou, Wei Xue

**Affiliations:** 1 College of Mechanical & Electrical Engineering, Wenzhou University, Wenzhou, 325035, P.R.China

## Abstract

Polymer-derived silicon oxycarbide (SiCO) presents excellent performance for high temperature and lithium-ion battery applications. Current experiments have provided some information on nano-structure of SiCO, while it is very challenging for experiments to take further insight into the molecular structure and its relationship with properties of materials. In this work, molecular dynamics (MD) based on empirical potential and first principle calculation were combined to investigate amorphous SiC_x_O_6/5_ ceramics. The amorphous structures of SiCO containing silicon-centered mix bond tetrahedrons and free carbon were successfully reproduced. The calculated radial distribution, angular distribution and Young’s modulus were validated by current experimental data, and more details on molecular structure were discussed. The change in the slope of Young’s modulus is related to the glass transition temperature of the material. The proposed modeling approach can be used to predict the properties of SiCO with different compositions.

Polymer-derived silicon oxycarbide (SiCO) presents excellent performance at high temperature^1^,^2^,^3^,^4^,^5^,^6^,^7^, moreover, it can give a lithium capacity of 1164 mAhg^−1^ with small volume change^8^, which makes it to be a promising anode material for LIBs. The unique nano-structure of SiCO is believed to be the key factor on its superior performance^4^,^9^. Graphene sheet (called free carbon) was found by Raman spectroscopy and in long-range order^10^,^11^. NMR (Nuclear magnetic resonance) and XPS (X-ray photoelectron spectroscopy) showed the existence of silicon centered tetrahedral with mixed bonds of carbon and oxygen^12^,^13^. A nanodomains model^4^ was proposed based on experimental results, the model composed with graphene cage-like network, clusters of silica that fill the cage and monolayer of SiC_n_O_4−n_ (Si-centered tetrahedral with mixed C/O bonds). Although experiments provide some information on the nano-structure of SiCO, the detailed molecular structure and how it influences the materials properties are still not clear, further study needs to be implemented based on atomistic scale structural and electronic properties.

Atomistic modeling and simulations are powerful tools for investigating various properties of materials, and was used in modeling of silicon^14^,^15^, SiC^16^, SiCN^17^,^18^ and SiCO^18^,^19^. Tomar^18^ studied the effects of temperature and morphology on the mechanical strength of SiCO and SiCN nanocomposites, as the morphologies were pre-designed in their models, the effect of composition on properties of materials were not studied. Kroll^19^ studied the structures and properties of stoichiometric silicon oxycarbide glasses with various compositions, the network structures were generated based on perfect Si–O and Si–C bonds, and carbon segregation was not fully reproduced.

In order to gain deeper insight in terms of free carbon and amorphous structure, in this work, molecular dynamics (MD) and first principle calculation were combined to investigate amorphous SiC_x_O_6/5_ ceramics. Melt-quench simulations were used to generate the initial structures, and then the structures were optimized further by ab initio molecular dynamics simulations. The obtained models successfully reproduce the structural features of SiCO, and the calculated properties are also consistent with current experimental results. To the best of our knowledge, this is the first study in which theoretical method is employed to study the role of composition on properties of amorphous SiCO.

## Model Construction

Melt-quench simulations based on classical molecular dynamics were used to generate the initial structures of SiCO. Tersoff potential^20^ and the parameters for SiO_2_^21^ and SiC^20^ were used to describe the particles interaction. The melt-quench procedure proposed in our previous study^17^ was used to generate the amorphous structures of SiCO from random distributed atoms, the temperature of system was adjusted by velocity scaling and canonical ensemble (NVT) using a Nosé–Hoover_thermostat. The melt-quench simulations were conducted by Lammps (Large-scale Atomic/Molecular Massively Parallel Simulator) code^22^.

The first principles calculations were then performed to optimize the initial structures further. The systems were annealed at 1500 K by 5 ps of NVT simulations, and then were quenched at a rate of 0.2 K/fs to 300 K with NPT ensemble. It is followed by a 5 ps of NPT simulation and a 5 ps of NVT simulation at 300 K for equilibration. Kinetic cut-off energies for the plane-wave expansion of wave function were set at 300 eV, the number of plane waves would change to maintain the fixed cutoff energy. The state of the electronic structure was described by the generalized gradient approximation (GGA)^23^. A 2 × 2 × 2 κ-points mesh size was employed to save enormous amount of computational time for ab initio molecular dynamics, this is validated to be sufficient for amorphous system.

Finally, all the amorphous structures were fully relaxed by geometry optimization of both lattice parameters and atomic position. The geometry optimization was conducted by using the conjugate gradient method under following conditions: residual force <0.01 eV/Å, convergence of energy change per atom <2 × 10^−6^ eV, stress <0.02 GPa. The first principles calculations according to the density functional theory (DFT) ultra-soft pseudo-potential method were performed by CASTEP code^24^,^25^ in Material Studio 5.5.

**Figure 1:**
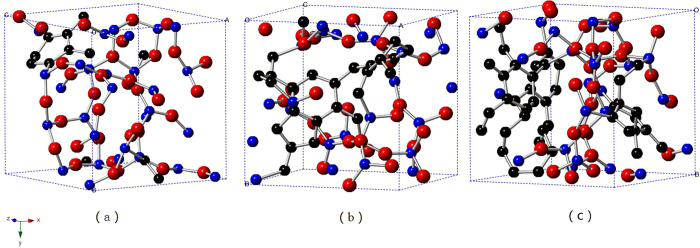
Generated amorphous SiCO structures. (**a**) SiC_2/5_O_6/5_, (**b**) SiCO_6/5_, (**c**) SiC_8/5_O_6/5_. (The Si, C and O atoms were presented as blue, black and red colors respectively).

Three SiCO structures with different compositions were studied in this work. SiC_x_O_6/5_ partitions as SiC_2/5_O_6/5_ and carbon, which refers to the stoichiometric glass (33.33 mol% SiC and 66.66 mol% SiO_2_) and excess carbon respectively. Three cases were studied here, 5:2:6, 5:5:6 and 5:8:6 for Si:C:O, in the order of increasing excess carbon. For each case, three independent structures were generated and analyzed to improve the statistics. As shown in Fig. 1(a), for the case with the lowest C content, i.e. the stoichiometric glass, there is no carbon network presented. With C content increases, two phases present in the SiCO structure, as shown in Fig. 1(b,c). They are carbon rich phase that like network expands to all the directions and the silica rich phase fill in the spaces of network accordingly, which results in a ‘nano-domain’ like structure.

## Results and Discussions

### Structural properties

**Figure 2:**
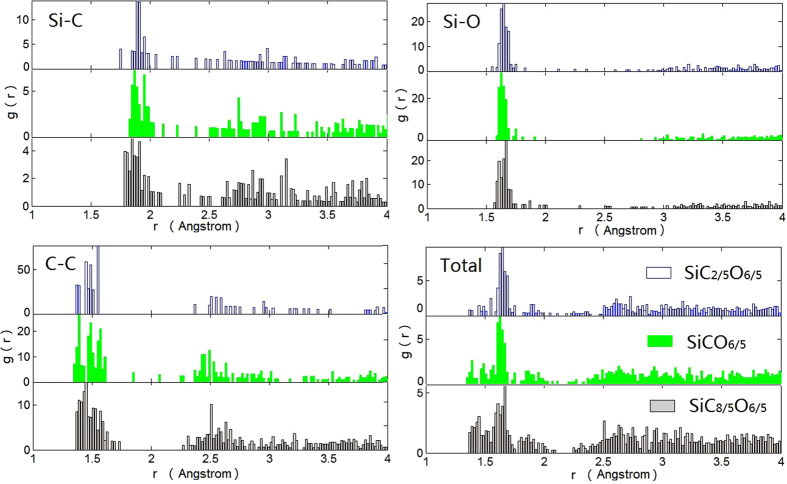
Radial distribution functions of the amorphous SiC_x_O_6/5_ structures.

The atomic correlations of the amorphous SiC_x_O_6/5_ structures are inferred by means of the radial distribution functions, as shown in Fig. 2. The bond length of Si–O, Si-C and C-C are determined by the sharp peaks observed at r_SiO_ = 1.62–1.64 Å, r_SiC_ = 1.84–1.89 Å and r_CC_ = 1.49–1.51 Å, correspond to the experimental result r_SiO_ = 1.62 Å, r_SiC_ = 1.88 Å and r_CC_ = 1.49 Å^2^,^26^. The first peaks of Si–C and C-C are slightly shift to lower values as carbon content increases, it relates to the formation of free carbon and structural change near the domains interfaces, as the enlargement of free carbon phase changes the Si-C and C-C correlations at its edge. The first peak and second nearest neighbor of total RDF are around 1.65–1.66 Å and 2.6–3.2 Å, which are comparable to the experimental results of 1.65–1.67 Å and 2.6–3.2 Å^26^. The first peak of total RDF is contributed by the SiO and Si-C bonds, and the small peaks for high carbon cases are related to the formation of free carbon. The second nearest neighbor distance for Si-C and C–C contribute to the peak of total RDF at 2.6–3.2 Å.

**Figure 3:**
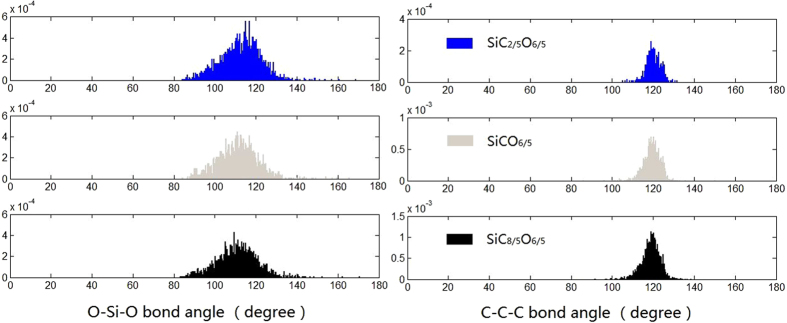
Angular distributions of the amorphous SiC_x_O_6/5_ structures.

Further information about the local structural is provided by the angular distribution, as shown in Fig. 3. There is a short range order defined by tetrahedron SiO_4_ which is characterized by O–Si–O angle with a clear peak at 112–118°. The average O-Si-O bond angle is getting smaller when carbon content increases, which is related to the presence of Si-C_3_O and Si-C_2_O_2_ tetrahedrons at the edge of free carbon phase, and this is in agreement with X- ray/ neutron diffraction^2^,^26^. The C-C-C angular distribution shows a main peak at 120°, it indicates the sp^2^ carbon character while also show a sp^3^ carbon character, which consists with the experimental conclusions on carbon character of SiCO^10^.

**Figure 4:**
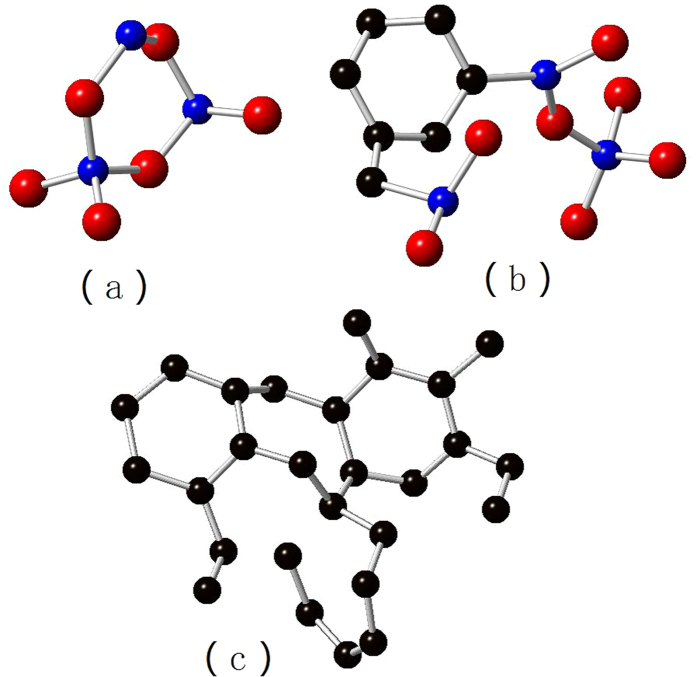
Typical molecular structure of the amorphous SiC_x_O_6/5_ structures.

Several typical molecular structures of the amorphous SiCO are shown in Fig. 4. The Si-O_4_ unit (Fig. 4a) dominants the Si-O region, and some of the silicon atoms form mixed bonds to carbon and oxygen atoms with a shape of silicon-centered tetrahedron (Fig. 4b), this is in agreement with experimental results^12^,^13^. Moreover, the carbon atoms at the edge of free carbon phase only connect to silicon atoms, and the silicon-centered mix-bond tetrahedrons only present at the interface of free carbon and Si-O region. This is still not validated, as indentifying molecular structure is still challenging for current experimental technologies. The free carbon (Fig. 4c) is clearly observed in a form of graphene, and its size tends to increase with higher carbon contents, which also consist with experiments^10^.

**Figure 5:**
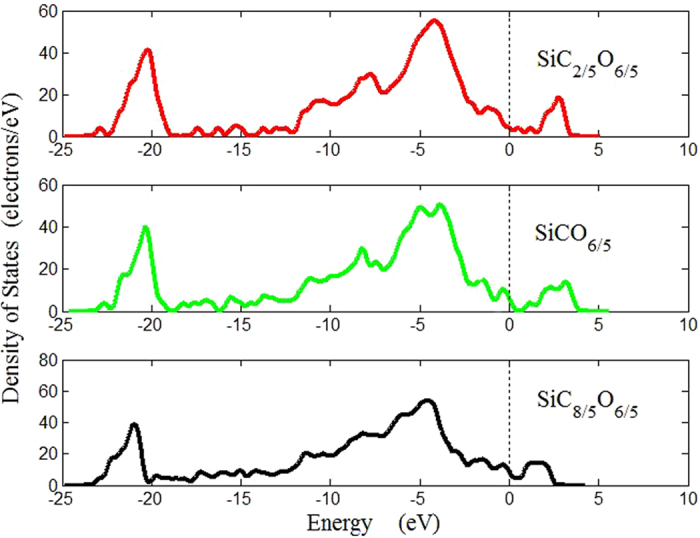
Density of states of the amorphous SiC_x_O_6/5_ structures.

In order to investigate the effect of carbon content on the electronic properties of the mixture, the density of state (DOS) of the amorphous SiC_x_O_6/5_ structures were calculated and are shown in Fig. 5. A small band gap is clearly visible especially for SiCO_6/5,_ which characterizes its semiconducting behaviors. The valence band is found to be wider with higher carbon content, the valence band DOS consists of bonding and nonbonding bands that are merged at the upper part of valence band, depending on different compositions. The band structures were also calculated and the generalized density functional theory (GDFT)^27^ was used to obtain corrected estimation of the band gap values. The band gaps are 0.428, 1.131, 0.311 eV for SiC_2/5_O_6/5_, SiCO_6/5_ and SiC_8/5_O_6/5_ respectively, it is concluded that the size of band gap can reach a maximum with a proper concentration of carbon, which is similar with the trend of SiCN^28^.

**Figure 6:**
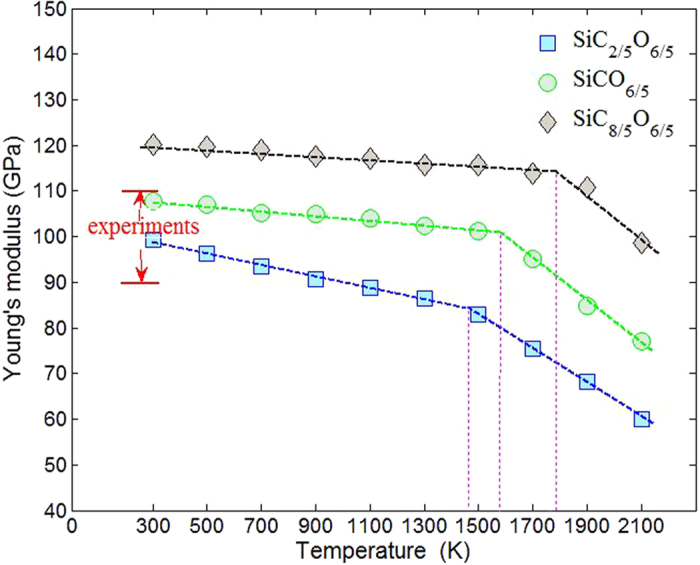
Young’s modulus of the amorphous SiC_x_O_6/5_ structures.

The mechanical properties were studied by classical molecular dynamics simulations of static tension test. Periodic boundary conditions were applied to all the three dimensions. In every 10 ps, the simulation box was displaced in z direction with strain of 0.002 and the structure was dynamically relaxed. The Young’s modulus (E) was then calculated by the obtained stress-strain curve. As shown in Fig. 6, the Young’s modulus of SiCO increase with an increased carbon content, as reported by nanoindentation test^29^. Within different compositions, it ranges from 99 GPa to 120 GPa, which is within the scope of 90–110 GPa in experiments^5^,^30^. There are changes in slope of Young’s modulus present at 1460–1785 K for all the compositions, which are very closed to the glass transition temperature of SiCO at 1573–1673 K from experiments^1^,^31^,^32^. The change in slope of temperature-dependent Young’s modulus was also used in experiments to indentify the glass transition temperature.

The Young’s modulus of SiCO decreases with temperature increases, it accords with common understanding of materials, however, the trend is different with Rouxel’s experimental results^1^. Their results showed that the Young’s modulus of SiCO increase slightly with increasing temperature. The discrepancy could be caused by several reasons, first of all, the ultrasonic technology was used to measure the changes in Young’s modulus in their study, which may lead to difference with our tensile-based approach. Another possible reason is the structure rearrangement of SiCO. According to the results of HRTEM^3^, annealing temperature exceeding 1100 °C for SiCO promote local decomposition to the escape of SiO, CO and CH_4_, and accompanied by the formation of SiC crystallites embedded in SiO_2_ matrix and in close to the carbon layers. At 1450 °C, the phase separation was observed in SiO_4_ and SiC_4_ rich region encapsulated by carbon, and the initially finely dispersed graphene layers grow and form thicker multi-layer carbon. These structure rearrangements may not be captured by our simulations because the limitations of empirical potential. Furthermore, the difference in composition is also a reason. Based on experiments and our simulations, the structures with higher carbon content generally show more stable Young’s modulus at high temperature.

## Conclusion

In this work, molecular dynamics and first principles calculations were combined to study the properties of silicon oxycarbide. The amorphous structures of SiCO with silicon-centered mix bond tetrahedrons and free carbon were successfully reproduced. The calculated radial distribution, angular distribution and Young’s modulus are validated by current experimental results, and more details on the molecular structure of SiCO are discussed. The valence band is found to be wider with higher carbon content, the calculated band gap can reach a maximum with a proper concentration of carbon. The change in the slope of Young’s modulus is related to the glass transition temperature of material. The proposed modeling approach is quite meaningful for further study of the unique properties of amorphous SiCO.

## Additional Information

**How to cite this article:** Liao, N. *et al*. Modeling of amorphous SiC_x_O_6/5_ by classical molecular dynamics and first principles calculations. *Sci. Rep.* **7**, 42705; doi: 10.1038/srep42705 (2017).

**Publisher's note:** Springer Nature remains neutral with regard to jurisdictional claims in published maps and institutional affiliations.
